# Combination therapy with anti-ErbB3 monoclonal antibodies and EGFR TKIs potently inhibits Non-small Cell Lung Cancer

**DOI:** 10.18632/oncotarget.1141

**Published:** 2013-07-21

**Authors:** Alessia Noto, Claudia De Vitis, Giuseppe Roscilli, Luigi Fattore, Debora Malpicci, Emanuele Marra, Laura Luberto, Antonio D'Andrilli, Pierpaolo Coluccia, Maria Rosaria Giovagnoli, Nicola Normanno, Luigi Ruco, Luigi Aurisicchio, Rita Mancini, Gennaro Ciliberto

**Affiliations:** ^1^ Dipartimento di Medicina Clinica e Molecolare, Sapienza Universita' di Roma, Italy;; ^2^ Dipartimento di Chirurgia “P. Valdoni”, Sapienza Università di Roma;; ^3^ Dipartimento di Medicina Sperimentale e Clinica, Università degli Studi di Catanzaro “Magna Graecia”, Catanzaro, Italy; ^4^ Takis s.r.l., Via di Castel Romano 100, 00128 Roma, Italy;; ^5^ BIOGEM scarl, via Camporeale, Ariano Irpino (Av), Italy;; ^6^ Azienda Ospedaliera S. Andrea, Roma, Italy;; ^7^ IRCCS Istituto Nazionale Tumori “Fondazione G. Pascale”, Napoli, Italy

**Keywords:** Lung cancer, primary cultures, TKIs, ErbB3, monoclonal antibodies

## Abstract

Personalized therapy of advanced non-small cell lung cancer (NSCLC) has been improved by the introduction of EGFR tyrosine kinase inhibitors (TKIs), gefitinib and erlotinib. EGFR TKIs induce dramatic objective responses and increase survival in patients bearing sensitizing mutations in the EGFR intracytoplasmic tyrosine kinase domain. However, virtually all patients develop resistance, and this is responsible for disease relapse. Hence several efforts are being undertaken to understand the mechanisms of resistance in order to develop combination treatments capable to sensitize resistant cells to EGFR TKIs. Recent studies have suggested that upregulation of another member of the EGFR receptor family, namely ErbB3 is involved in drug resistance, through increased phosphorylation of its intracytoplasmic domain and activation of PI3K/AKT signaling. In this paper we first show, by using a set of malignant pleural effusion derived cell cultures (MPEDCC) from patients with lung adenocarcinoma, that surface ErbB3 expression correlates with increased AKT phosphorylation. Antibodies against ErbB3, namely A3, which we previously demonstrated to induce receptor internalization and degradation, inhibit growth and induce apoptosis only in cells overexpressing surface ErbB3. Furthermore, combination of anti-ErbB3 antibodies with EGFR TKIs synergistically affect cell proliferation *in vitro*, cause cell cycle arrest, up-regulate p21 expression and inhibit tumor growth in mouse xenografts. Importantly, potentiation of gefitinib by anti-ErbB3 antibodies occurs both in de novo and in ab initio resistant cells. Anti-ErbB3 mAbs strongly synergize also with the dual EGFR and HER2 inhibitor lapatinib. Our results suggest that combination treatment with EGFR TKI and antibodies against ErbB3 should be a promising approach to pursue in the clinic.

## INTRODUCTION

Lung cancer is the most commonly diagnosed type of cancer and the primary cause of cancer-related deaths worldwide [1,2]. Lung cancer is associated with poor prognosis because disease remains largely asymptomatic for a long period of time [3]; a significant proportion of patients are diagnosed at an advanced stage when surgery is no longer an option and often when metastases have already diffused to other organs. The most common type of lung cancer is non-small cell lung cancer (NSCLC) which accounts for approximately 80-85% of all cases [4]. In the last ten years the introduction of targeted therapies has positively impacted upon the prognosis of a subsets of NSCLC patients carrying either mutations in EGFR, EML-ALK translocations or KRAS mutations, for which specific inhibitors of the relevant kinases have been developed [5,6].

The epidermal growth factor receptor (EGFR) is a transmembrane protein with an extracellular ligand binding domain and an intracellular domain possessing intrinsic tyrosine kinase activity. EGFR belongs to a family of four transmembrane receptors (ErbB2/HER2, ErbB3/HER3 and ErbB4), commonly called also ErbBs which, upon ligand-driven homo or heterodimerization and subsequent activation of their receptor-associated tyrosine kinase domains, stimulate downstream signaling cascades leading to cell proliferation, motility and survival [7,8]. ErbBs have been linked for the past twenty years to the process of tumorigenesis and therefore the object of intense studies directed to the development of their inhibitors as cancer therapeutics. Indeed EGFR was found to be overexpressed in several types of tumors including lung carcinomas [9]. In particular in different series of NSCLCs overexpression of EGFR ranging from 45 to 90% was reported [10,11]. Since inhibition of EGFR by specific blocking agents causes growth inhibition in in vitro and in vivo tumor models, this justified the initial development of TKI inhibitors gefitinib and eroltinib [5,12]. However it was soon discovered that neither the expression level nor constitutive phosphorylation of EGFR predict clinical responses to TKI inhibitors [13]. Instead compelling evidence was provided showing that only the presence of mutations within EGFR can distinguish responders from non-responders. It is now well established that specific genetic alterations in exons 18, 19 and 21 of the EGFR gene, which are found in approximately 10-15% of patients diagnosed with NSCLC, are predictive of response to TKIs. This implies the necessity to use these compounds in strictly personalized approaches to the therapy of lung cancer. Several Phase III clinical trials have shown statistically significant superiority to standard chemotherapy in terms of response rate, progression-free survival and quality of life in patients with NSCLC across all therapy lines. In light of these studies TKIs like gefitinib can now be considered as the standard first-line treatment of patients with advanced NSCLC harboring activating EGFR mutations [5,14-19].

In spite of these positive aspects, the impact of EGFR TKIs on overall survival remains marginal in patients with advanced disease. This is generally believed to be due to the rapid development of drug resistance. Several are the mechanisms identified to be responsible of resistance [20], the most frequent being the occurrence of a secondary so called “gatekeeper” mutation T790M in EGFR which accounts for about 50% of cases [21-23]. Likewise, amplification of cMet was described to occur in cells resistant to TKI treatment and cMet transphosphorylation of ErbB3 was shown to be a mechanism whereby resistant cells can circumvent blockade of EGFR activity [24-25]. Moreover, suboptimal pathway inhibition by tyrosine kinase inhibitors (TKIs) was shown to result in a compensatory shift in ErbB3 activation [26]. In all these cases the central involvement of ErbB3 in the development of resistance has been suggested.

ErbB3 has been disregarded for several years as a cancer target, although the elevated expression of this receptor in several human cancers led in early times to postulate its involvement in tumor progression [27-29]. This low interest in ErbB3 was also due to the lack of detectable mutations in cancer samples and the absence of a strongly active tyrosine kinase in its intracellular domain [30]. However, during the past 5 years a mounting number of evidences have been accumulated pointing to a key role of this receptor in tumorigenesis and cancer progression and, above all in the establishment of resistance to therapies [26, 30]. These evidences have triggered major efforts towards the development of anti-ErbB3 therapies. Because this receptor is devoid of strong intrinsic kinase activity, the major strategy in this case is the generation of monoclonal antibodies directed against the receptor. Some of these have already entered clinical development [31].

Our group has recently generated a set of anti-ErbB3 monoclonal antibodies. We have previously shown that two antibodies, named A3 and A4, displaying low nM affinity for the receptor, are able to block ligand induced receptor phosphorylation and downstream AKT signaling in a variety of cancer cells and to efficiently inhibit tumor growth in xenograft models [32].

In this paper we demonstrate the ability of anti-ErbB3 antibodies to sensitize cells resistant to EGFR TKIs to these drugs both in an established lung cancer cell line and in primary cultures from malignant pleural effusions of lung adenocarcinoma patients, and that this effect correlates with the expression levels of ErbB3. Furthermore we started to address the mechanism responsible for synergism.

## RESULTS

### High surface ErbB3 expression correlates with AKT phosphorylation in lung adenocarcinoma primary cultures

**Figure 1:**
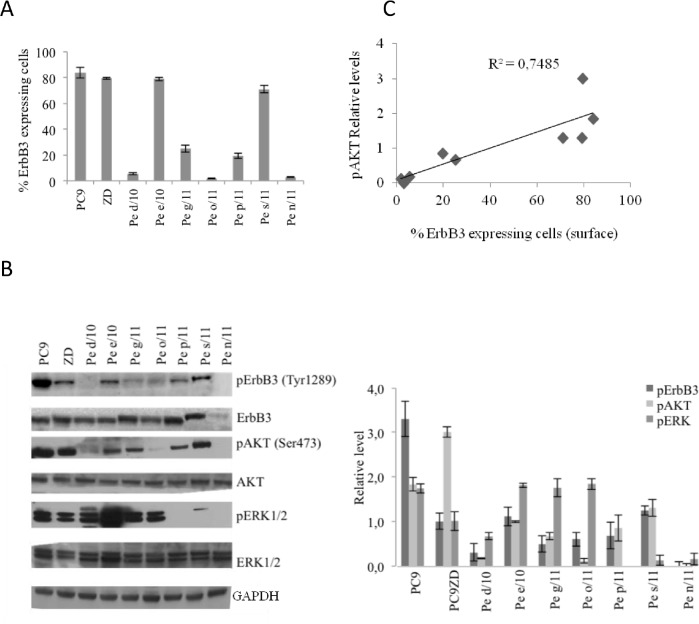
ErbB3 expression correlates with enhanced AKT signaling in primary and stable lung cancer cells (a) Percentage of positive cells expressing surface ErbB3 was determined by FACS analysis in the indicated cell lines. Data represent the mean ± SD of three independent experiments. (b) Western Blot analysis of basal level of ErbB3 and pErbB3 and its downstream signalling in 7 representative primary MEPDCCs and stable cell lines PC9 and PC9ZD. At the right panel relative densitometry was evaluated. Data represent the mean ± SD of three independent experiments. (c) Graphic correlation between pAKT and surface ErbB3. Spearman's correlation index=0.88, p=0.003. pAKT was also strongly correlated with pErbB3 (Spearman's correlation index=0.82, p=0.011).

In order to identify a lung adenocarcinoma cell system suitable to study the efficacy of our anti-ErbB3 monoclonal antibodies we screened several cells for surface ErbB3 expression. We have previously described an efficient procedure to establish in vitro primary cell cultures from Malignant Pleural Effusions of patients affected by adenocarcinoma of the lung [33]. Using this protocol a collection of MPEDCC (Malignant Pleural Effusion Derived Cell Cultures) suitable to investigate lung cancer heterogeneity and response to therapies was obtained. We decided to analyze the expression of ErbB3 in a representative set of samples. We first measured surface expression of ErbB3 by cytofluorimetry (Figure 1a, Table 1). To this purpose we utilized 7 MPEDCC and as control a stable lung Adeno Ca cell lines, PC9 which is highly sensitive to gefitinib (IC_50_ 0,015 μM) for the presence of exon 19 deletion, and its gefitinib resistance subclone PC9ZD, which harbors the gatekeeper T790M (Table 1, Supplementary 1) [34,35]. Results showed great heterogeneity in the expression level of ErbB3 on cell surface, which allowed us to assign samples to three groups having high (>50%), intermediate (>10 <50%) and low (<10%) ErbB3 levels respectively.

**Table 1:** Gefitinib sensitivity of primary and stable lung cell lines IC50 values were obtained for gefitinib with MTT assays in PC9, PC9ZD cell lines and in seven primary cell cultures. Mutational analysis for exon 19, exon 21 was performed. Secondary EGFR T790M mutation was also evaluated.

Cell culture	IC50 Gefitinib μM	Exon 19 Del	L858R (ex21)	L861Q (ex21)	T790M
PC9	0,015	MUTATED	WT	WT	WT
PC9ZD	12,2	MUTATED	WT	WT	T790M
Pe d/10	10	WT	WT	WT	WT
Pe e/10	9,7	WT	WT	WT	WT
Pe g/11	10	MUTATED	WT	WT	WT
Pe o/11	33,1	WT	WT	WT	WT
Pe p/11	12,3	MUTATED	WT	WT	T790M
Pe s/11	3,4	MUTATED	WT	WT	T790M
Pe n/11	12,3	WT	WT	WT	WT

WB analysis (Figure 1b) confirmed high variability in the expression of total ErbB3, which however was not always corresponding to the surface expression of the receptor, thus suggesting that not in all cases the receptor is efficiently exposed to the cell surface and therefore likely functional. Interestingly however, we found that high surface expression of ErbB3 correlated both in primary cells and in the PC9/PC9ZD cell lines to elevated pAKT levels (Spearman's correlation coefficient=0.88, p=0.003), suggesting that activation of the PI3K/AKT survival pathway is strictly associated to surface ErbB3 expression and activation by its ligand (Figure 1c). In addition, the heterogeneity of the primary tumor cell lines was also reflected in the variability of activated MAPK pathway.

### Anti-ErbB3 monoclonal antibodies affect HRG signaling and induce apoptosis in MPEDCC lung adenocarcinoma cultures

The high expression of ErbB3 on the surface of a subset of lung adenocarcinoma cell cultures led us to explore the effect of anti-ErbB3 monoclonals directed against the extracellular domain of the receptor. We previously reported the generation of two anti-human ErbB3 monoclonals, A3 and A4, which bind the receptor with nM affinity and are able to efficiently compete for ligand binding and to inhibit AKT signaling [32]. These two monoclonals recognize different epitopes, both of them strongly inducing receptor internalization and degradation [36].

**Figure 2:**
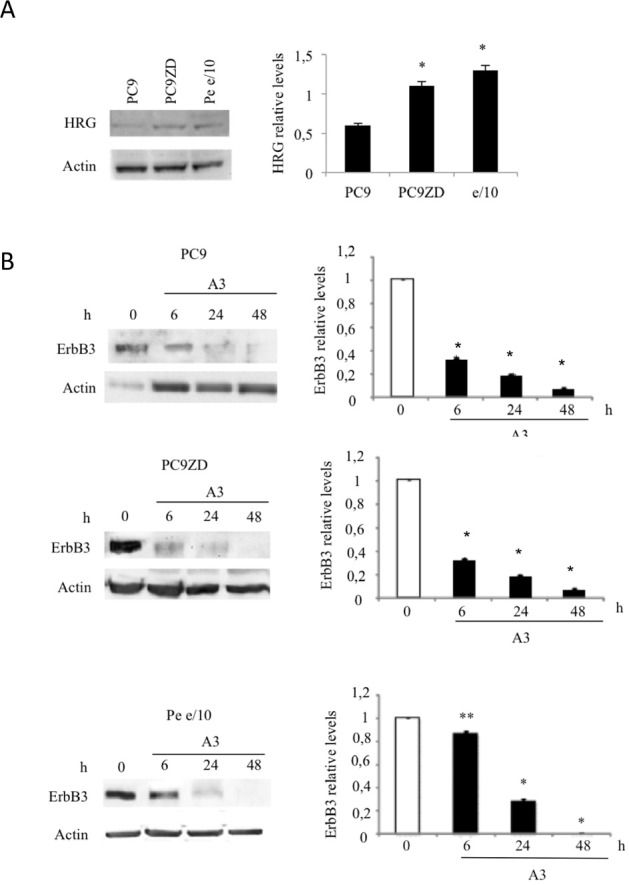
A3 mAb induces a time dependent down-modulation of ErbB3 (a) Representative Western blot of Heregulin expression in PC9, PC9ZD and Pe e/10 cell lines and relative densitometry were illustrated. (b) Cell cultures were treated at the indicated times with A3 at 50 μg/ml. Total ErbB3 was evaluated by western blot. Data are the mean ± SD of three independent experiments. *p<0.01, **p<0.05 versus untreated cells.

**Figure 3:**
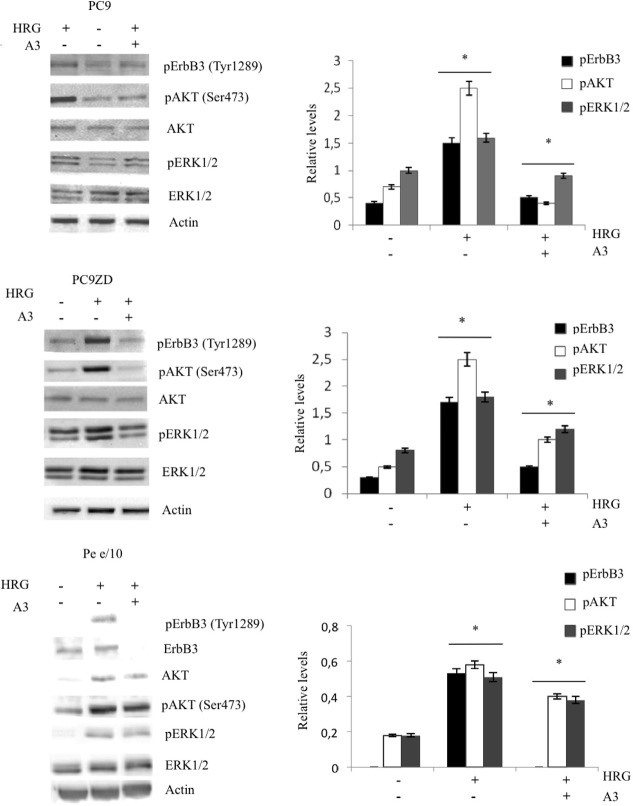
A3 inhibits HRG-induced signaling Phosphorylation of AKT, ERK1/2 and ErbB3 was determined in PC9, PC9ZD and Pe e/10 treated with 50 μg/ml of A3 for 6 hrs and stimulated with 50 ng/ml of HRG for 10 min. At the right panel densitometries were calculated for phosphorylated proteins with respect to each total proteins. Data shown represent the mean ± SD of three independent experiments. *p<0.01 versus untreated cells.

In order to verify whether one of these monoclonals, named A3, was able to inhibit ErbB3 signaling in MPEDCCs, we evaluated both ErbB3 downmodulation and signaling inhibition by WB analysis in PC9, PC9ZD and Pe e/10 as a representative MPEDCC with high ErbB3 surface expression. To confirm an active ErbB3 signaling and if this was due to an autocrine ligand-receptor loop, we also analyzed heregulin (HRG) expression by western blotting (Figure 2a). A3 was able induce downmodulation of the receptor in a time dependent manner (Figure 2b), with a noticeable effect in the Pe e/10 cell culture. Moreover, we observed that treatment for 24 hr with A3 inhibited HRG-induced phosphorylation of both ErbB3, AKT and, to a minor extent also of pERK 1/2 (Figure 3).

### Anti-ErbB3 monoclonals inhibit proliferation and induces apoptosis in lung cancer cells

**Figure 4:**
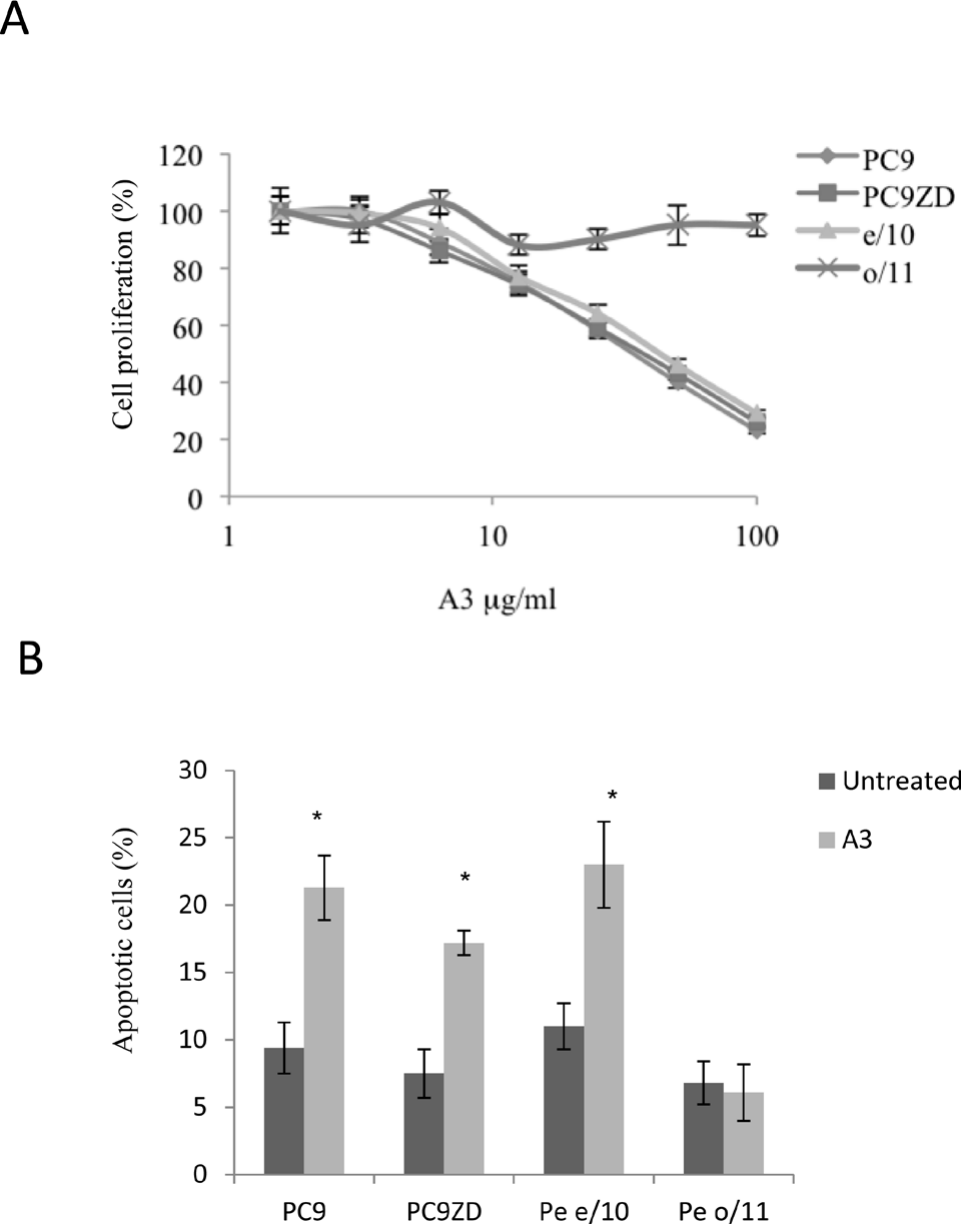
A3 affects proliferation and induces apoptosis in high surface expressing ErbB3 cells (a) Clonogenic assay was performed in different cell cultures with different doses of A3, as described in Materials and Methods. (b) Apoptosis induction was evaluated by FACS analysis using Annexin V staining after 72 hr of treatment with 50 μg/ml of A3. Results are the mean ± SD of three independent experiments.* p<0.01 versus untreated cells.

On the basis of previous results we next assessed cell growth inhibition in highly surface ErbB3 expressing cells, PC9, PC9ZD, and Pe e/10 using a clonogenic assay. We observed significant inhibition by A3 (Figure 4a, Supplementary 2) in all analyzed cell lines. Moreover, on a MPEDCC in which ErbB3 membrane expression was low, namely o/11, the mAb did not impact cell proliferation thus indicating that the effect was specific only for cells expressing high surface levels of the receptor and with concomitant high basal level pAKT as previously shown (Figure 4a, Supplementary 2, and Figure 1). In order to provide further insights into the biological effect of A3 we evaluated apoptosis induction by flow cytometry in the cell cultures in which A3 inhibited the signaling. Consistently, in the cell cultures analyzed we observed an increased percentage of cells that underwent apoptosis after 72 hours of treatment, while the cell line with low ErbB3 expression did not, as confirmed by Annexin V staining (Figure 4b and Supplementary 3).

### Anti-ErbB3 monoclonals potentiate the effect of gefitinib in a subset of lung cancers

Analyzing the presence of the most frequently EGFR mutations, in particular gefitinib sensitizing mutations, we determined that exon 19 deletion was present in a significant proportion of the cell lines. Interestingly, when our collection of MPEDCC was tested for sensitivity to gefitinib, we observed that the majority of them were resistant (Table 1). Plasma trough concentration of the drug achieved during therapy is approximately 1 mM, therefore this is generally considered the resistance threshold [36]. However, we found that only two out of six cell lines contained the T790M mutations. We thus wondered whether A3 could potentiate the effect of gefitinib in lung cancer cells with spontaneous or acquired resistance to this TKI.

**Figure 5:**
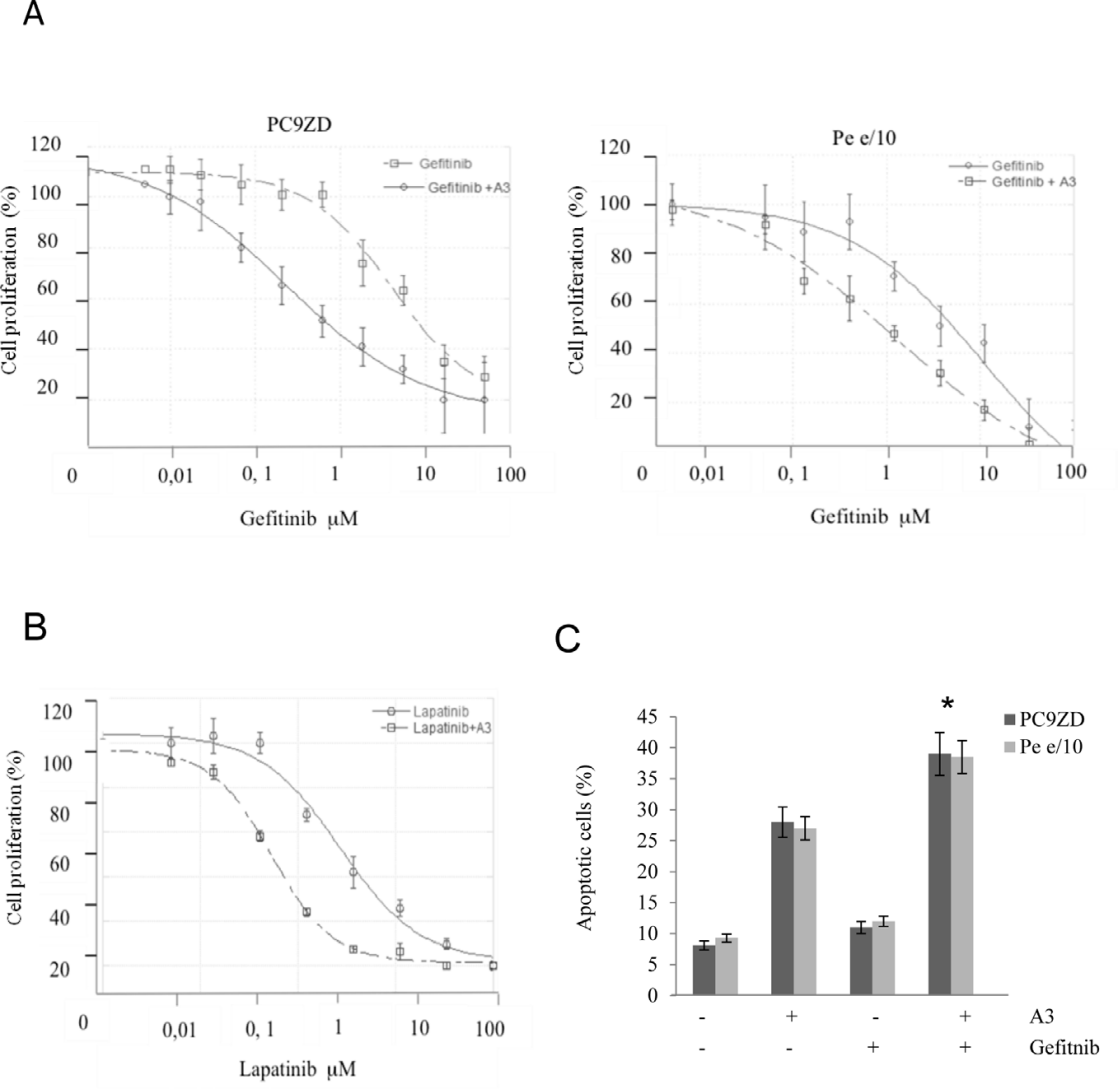
A3 potentiates the effect of TK inhibitors in gefitinib resistant lung cancer cell cultures both in a clonogenic assay and in apoptosis induction (a) PC9ZD and Pe e/10 cell cultures were incubated with various concentration of gefitinib and/or A3 at 25 μg/ml for 10 days in a clonogenic assay and analyzed as described in Material and Methods. Results are the mean ±SD of three independent experiments. (b) Clonogenic assay was performed on PC9ZD incubated with lapatinib and or/A3 at 25 μg/ml for 10 days as described above. Results are the mean ±SD of three independent experiments. (c) Apoptosis induction was evaluated in PC9ZD and Pe/e10 cell cultures after 72 hrs of treatment with A3 at 50 μg/ml and/or 1 μM gefitinib. Apoptosis was analyzed using Annexin V as described in Material and Methods. Results are the mean of three independent experiments ±SD. *p<0.01 in respect of untreated cells.

To assess the effect of anti-ErbB3 monoclonals as sensitizers to gefitinib, we focused on MPEDCC Pe e/10, which has high expression of ErbB3/ high resistance to the drug (IC50=9,3 μM) and PC9ZD resistant cell line which contains the Del exon and harbors the T790M mutation (IC50 = 14,4 μM Supplementary Figure 2). In clonogenic assays we observed an increased growth inhibition in cell cultures treated for 10 days with A3 in combination with gefitinib, as compared to treatment with gefitinib alone, both in PC9ZD line and in e/10 (Figure 5a, Supplementary Figure 4, Table 2). Similar results were obtained upon co-treatment of cells with the other approved EGFR TKI erlotinib (Supplementary Figure 5) and with the dual EGFR/Her2 kinase inhibitor lapatinib (Figure 5b).

**Table 2:** A3 increases sensitivity to gefitinib IC50 values for gefitinib were calculated in clonogenic assays in PC9ZD and Pe e/10 cell cultures with or without the presence of 25 μg/ml of A3. The increase sensitivity was calculated as ratio.

Cell line	IC50 Gefitinib	IC50 Gefitinib +A3	Ratio
PC9ZD	4 μM	0,22 μM	18
e/10	10 μM	1,24 μM	8

This potentiating effect was confirmed by the assessment of apoptosis in resistant PC9ZD and Pe e/10 cell cultures. Apoptosis induction was higher in cells treated with the combination of A3 and Gefitinib, with respect to single treatments (Figure 5c).

**Figure 6:**
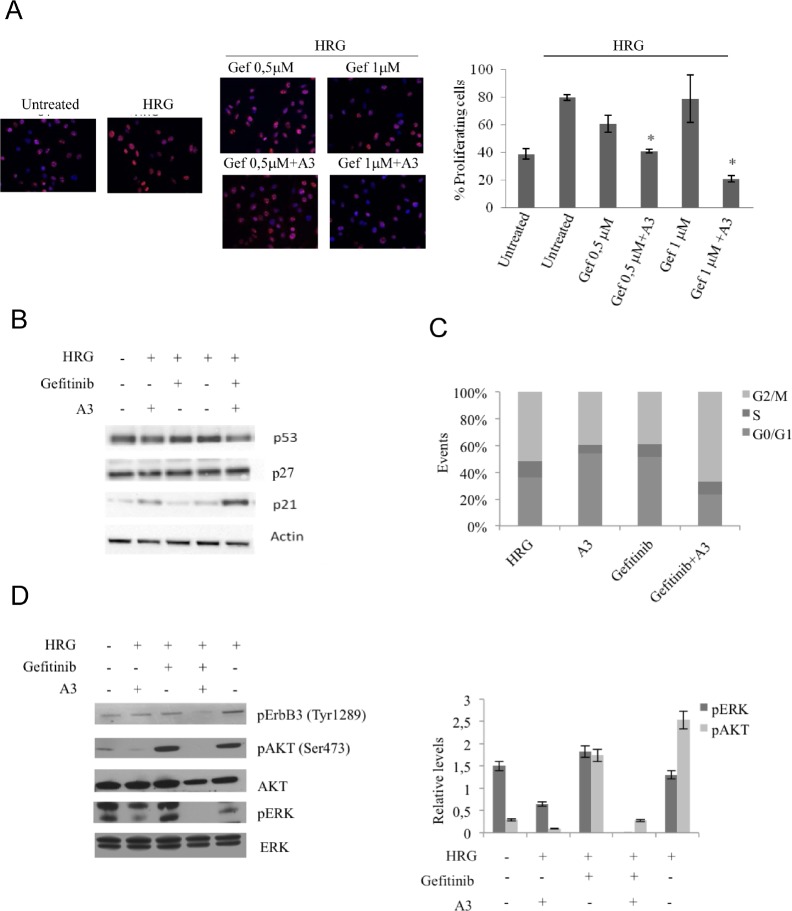
The combination A3 plus gefitinib reduces the proliferation, impairs cell cycle and reduces pAKT and pERK signaling (a) PC9ZD cells were pre-treated with 50 μg/ml A3, then incubated with HRG for 24 hrs and stained with anit-Ki67 antibodies to identify cycling cells. Quantitative analysis of the percentage of cells presenting Ki67-positive nuclei was performed as reported in Material and Methods. Values are the mean ±SD. *p<0.05 versus HRG treated cells. (b) PC9ZD cells were treated for 24 hrs with indicated compounds and p53, p27 and p21 protein level were evaluated by western blotting. (c) Cell cycle analysis was evaluated by FACS analysis on PC9ZD treated with A3, gefitinib or the combination. (d) Phosphorylation of AKT and ERK1/2 was evaluated by Western blotting in PC9ZD cell line treated as above. For densitometric analysis results are expressed as mean values ± SD from three independent experiments.

Moreover, in resistant PC9ZD we observed that HRG-induced proliferation as measured by Ki67 staining was dramatically impaired by the combination A3 plus gefitinib (Figure 6a).

When we investigated the expression of the principal cell cycle regulators, we saw an increased upregulation at the protein level of p21 in A3 treated cells, while the only gefitinib was not able to elicit this effect both in PC9ZD and Pe e/10. This result was more evident in cells treated with the combination, indicating a possible role of A3 monoclonals to induce p21 and consequently determine an arrest in the cell proliferation (Figure 6b and Supplementary Figure 6a). The p21 induction, moreover, was independent of p53 upregulation as observed by western blot and was regulated in a post-transcriptional manner (Supplementary Figure 6b). Moreover, cell cycle analysis showed an arrest of cells in G2/M checkpoint when cells were treated with both gefitinib and A3 (Figure 6b).

Interestingly, analyzing the signaling pathways, the combination resulted in a greater inhibition of ERK HRG-induced phosphorylation, thus confirming that A3 together with gefitinib are able to cooperate in repressing the AKT and ERK pathway (Figure 6c).

### Anti-ErbB3 antibodies potentiate the effect of gefitinib in vivo

**Figure 7:**
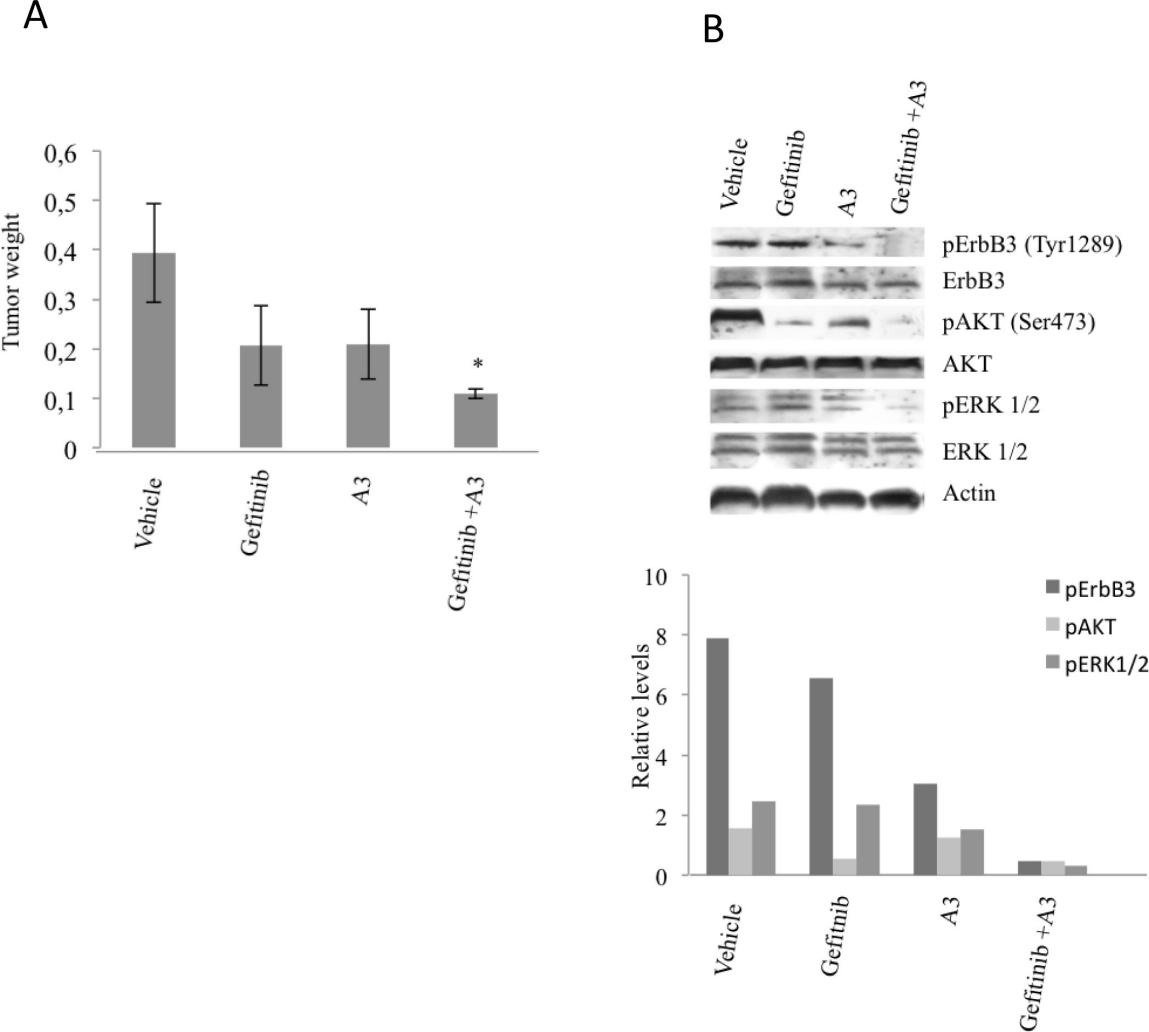
A3 increases the efficacy of gefitinib in vivo NOD/SCID mice xenografted with Pe e/10 primary cultures were treated with either gefitinib (100 mg/Kg) or A3 (20 mg/Kg) alone or with the combination of both. After 4 weeks mice were sacrificed and tumors weight were determined. *p<0.05 versus vehicle.

To determine if A3 antibody, blocking ErbB3 receptor activity, is able to potentiate *in vivo* the effect of Gefitinib on resistant tumor, xenograft tumors from Pe e/10 primary culture were established in immunodeficient mice. Pe e/10 primary culture carries wild type EGFR receptor and is highly resistant to Gefitinib treatment (Table 2). Moreover Pe e/10 cells express high levels of ErbB3 receptor which is also exposed on the cell membrane of most of the cells (Figure 1, Table 1). Secondary xenografts were established by serially passaging xenograft obtained by s.c. injections in NOD/SCID mice. Once tumor reached 100 mm^3^, mice were randomized and allocated in the following experimental groups: vehicle treated, gefitinib treated (100 mg/10ml/kg, p.o., daily, 5 days/week), A3 treated (20 mg/10 ml/Kg, i.p., once per week), and combination of gefitinib and A3. Tumor growth was initially followed by caliper, but we found some inconsistent values during the course of the experiment due to the preference of this tumor to grow toward the peritoneum instead of expanding subcutaneously. Treatments were continued for four weeks and mice were then sacrificed to determine if an effect was appreciable on tumor masses. After harvesting, tumor weight was determined and we found that co-treatment had a greater impact on tumor growth. Gefitinib or A3 monotherapy treatment, reduced tumor masses of about 60%. However, these results were not statistically significant in comparison with vehicle treatment alone. The combination of A3 and Gefitinib was more efficacious in reducing tumor mass (70% inhibition vs vehicle treated group, p< 0.05) as compared to monotherapies (Figure 7a). To determine the consequence of treatments on ErbB3 pathway, total cell extracts from tumor samples were analyzed by western blot. The results are shown in Figure 7b and indicate a strong impairment of pAKT and pERK signaling when A3 and gefitinib were administered in combination. These data therefore suggest that *in vivo* double inhibition of ErbB3 and EGFR can achieve stronger antitumoral effects.

## DISCUSSION

Therapy of NSCLC with first generation small molecule EGFR kinase inhibitors, gefitinib and erlotinib, is severely limited by two main factors: first, the poor sensitivity to TKIs of tumor cells expressing wild type forms of the receptor [14-19]; second the emergence of drug resistance in virtually all tumors bearing EGFR mutations initially sensitive for the presence of either exon 19 deletions or exon 21 mutation L858R [21-23,38]. In this context it is important to identify factors that contribute to EGFR-induced tumor cell growth because their targeting may help sensitizing cells to the activity of TKIs.

*De novo* resistance to TKIs has been the subject of intense studies over the past years. These have led to the identification of multiple mechanisms, among them the most frequent ones are either the occurrence of the secondary gatekeeper mutation T790M mutation in the EGFR intracytoplasmic domain or cMET amplification. These findings have fostered new approaches directed to the development of second generation irreversible EGFR inhibitors [19,39], or also to the clinical development of cMET inhibitors [40]. In virtually all *de novo* resistant NSCLC tumors the ErbB3 receptor is strongly phosphorylated [23,25,41]. ErbB3 does not have an intrinsic tyrosine kinase activity; however it can be very efficiently phosphorylated *in trans* by cMET or by other RTKs such as for example ErbB2 or ErbB4 [42]. ErbB3 strongly cooperates with the other members of the ErbB family in the activation of intracellular pro-survival signaling due to the presence of several tyrosine residues in its intracytoplasmic domain which, upon phosphorylation, become high affinity docking sites for the catalytic subunit of PI3K. Based on these evidences ErbB3 may represent a key node to co-target in order to potentiate the activity of EGFR TKIs. The cooperation between EGFR and ErbB3 may be playing an important role not only in cells which acquire *de novo* resistance to gefitinib and erlotinib but also in primary resistant cells bearing only wild-type EGFR. In this paper we have investigated this aspect with the use of two distinct tools, namely MPE-derived primary cultures of NSCLC and anti-ErbB3 antibodies recently generated in our laboratory.

MPE-derived tumor cells propagate in culture at high efficiency and represent cells with high propensity to metastasize. Using seven independent cultures it was possible to group them in high ErbB3 surface expressors (>70%), intermediate ErbB3 surface expressors (>20% < 70%) and low ErbB3 surface expressors (<20%). Interestingly, both high and intermediate surface ErbB3 expression corresponded to elevated levels of pAKT, which suggests that surface expression of ErbB3 above a certain threshold may help promoting NSCLC survival independently of the presence of a wild-type or of a mutated form of EGFR (see Table 1). Our collection of primary MPE-derived cultures present an elevated frequency of mutations in EGFR (3 out of 7) which is much higher than the reported frequency of EGFR mutation in NSCLC. In two out of four cases (g/11 and s/11) histological sections of the primary tumors were available, which allowed to carry out EGFR sequencing. Surprisingly, in these two cases no Exon 19 deletion was detected in contrast with the cell cultures data (data not shown). At the moment we are unable to explain this discrepancy. One possibility is that a small proportion of EGFR mutated cells is already present in the primary tumor, below the detection limit of the sequencing analysis and these cells are selected *in vivo* because of their increased fitness to metastasize in the pleura, and/or also *in vitro* by the cell culture conditions used.

ErbB3-driven phosphorylation of AKT may contribute to low sensitivity to TKIs. In order to address this aspect we focused our attention on one of the high surface expressing cells, Pe e/10 and as controls on two stable NSCLC cell lines, the gefitinib sensitive PC9 and its resistant counterpart PC9ZD bearing the T790M mutation [34,35]. Interestingly, both PC9ZD and Pe e/10 which are resistant to gefitinib express higher levels of the ligand heregulin than PC9. We first observed in these cells that the anti-ErbB3 antibody A3 was able to induce strong ErbB3 downregulation as previously shown in melanoma cells and that the antibody was able to decrease, albeit to a variable extent, heregulin-induced pErbB3, pAKT and pERK signaling [36]. The same antibody was able to affect cell proliferation and to induce apoptosis, when used at doses above 10 μg/ml. Importantly antibody efficacy correlated with surface ErbB3 expression because the same antibody was unable to affect cell growth and apoptosis of one of the low surface ErBB3 expressing cell lines Pe o/11.

When we went to analyze the potential synergy of the anti-ErbB3 antibody A3 with gefitinib both in acquired (PC9ZD) and EGFR wild-type (Pe e/10) resistant cells, we observed an approximate 20-10 fold sensitization of cells to the TKI and a significant shift to the left of the dose-response curve. The ability of A3 to potentiate the effect of gefitinib in EGFR mutated cells (PC9ZD) was confirmed in a variety of assays including short term cell proliferation (Ki67), apoptosis and cell cycle. This latter analysis showed that combinatorial treatment of PC9ZD cells with A3 and gefitinib resulted mainly in G2/M arrest, in line with the strong induction of p21. Interestingly, A3 treatment caused a pronounced induction of p21 in the absence of increased p53 levels, which was even further potentiated by co-treatment with gefitinib. It will be interesting to further analyze the mechanism of p21 induction by anti-ErbB3 antibodies in NSCLC and if this is due to activation of an intracellular pathway leading to increased p21 stabilization. It is also important to point out that potentiation of TKI activity was not only observed with gefitinib but also with the other first generation EGFR TKI erlotinib and, more intriguingly with lapatinib. This last observation suggests that also in the presence of simultaneous inhibition of both EGFR and HER2 kinases, ErbB3 is still able to signal and to promote cell growth and survival, probably via trans-phosphorylation by other kinases and that the only approach to fully abolish its activity is through the use of monoclonal antibodies capable to induce its internalization and degradation. Hence this finding further underscores the necessity to co-target this receptor in order to obtain maximal therapeutic efficacy of TKI inhibitors in lung cancer.

Finally, potentiation of gefitinib by the anti-ErbB3 antibody A3 was confirmed in an *in vivo* xenograft model with Pe e/10. These cells, when implanted subcutaneously, show a high propensity to invasive growth in the underlying tissues. Necropsy analysis of mice implanted with Pe e/10 showed significant tumor growth inhibition only upon co-treatment, which also resulted in increased inhibition both in AKT and ERK phosphorylation.

In conclusion, antibodies against ErbB3 may become new tools in our repertoire of anticancer agents for the therapy of NSCLC because of their ability to potentiate the effect of EGFR inhibitors. It is important to underline that use of these agents requires a rational approach based on the evidences presented in this paper. First, anti-ErbB3 antibodies will be effective only in the subset of NSCLC which express surface levels of the receptor above a certain threshold which we have also shown to correspond to increased AKT pathway activation levels. Hence surface ErbB3 may be considered a predictive marker of efficacy if appropriately validated in a higher number of cases. Furthermore, also in these cases ErbB3 should not be considered the main driver of tumor growth but rather a co-promoting factor. For this reason, anti-ErbB3 antibodies may not result to be highly efficacious by themselves as single agents, but rather to act as potentiators of small inhibitors of EGFR or of other RTKs. In light of these considerations, extreme care should be played in the clinical development of anti-ErbB3 agents in order to fully exploit their potential.

## MATERIALS AND METHODS

### Antibodies and Reagents

Gefitinib was purchased from Santa Cruz Biotechnology, Lapatinib and Erlotinib were from Selleck Chemicals. A3 monoclonals were generated as previously described.^24^ Antibodies against phospho-ErbB3, phospho-Akt, phospho-ERK1/2, anti-ERK, anti-AKT were purchased from Cell Signaling Technology. Anti-EGFR, anti-ErbB3, anti-p21, anti-p27 and anti-p53 were obtained from Santa Cruz Biotechnology. Anti GAPDH and Actin were purchased from Sigma Aldrich. Anti HRG was from Santa Cruz Biotechnology. The rabbit anti-Ki67 polyclonal antibodies were from Zymed Laboratories. HRP-conjugated secondary antibodies were from Sigma Aldrich. Texas Red-conjugated goat anti-rabbit IgG were from Jackson Immunoresearch Laboratories. DAPI was purchased from Sigma. Human HRG-1-β1 (HRG) was purchased from R&D Systems.

### Cell lines and primary cell cultures

PC9 and PC9ZD lung cancer cell lines were a kind gift of Dr Nishio Kazuto. PC9 and PC9ZD were cultured in RPMI-1640 (Gibco) supplemented with 10% Fetal Bovine Serum (Gibco), penicillin and streptomycin 100 U/ml and 100 μg/ml respectively (Biowest), in a humidified atmosphere of 5% CO_2_ at 37°C. Primary cell cultures were obtained from malignant pleural effusions of patients affected by NSCLC. Protocol of MPE-derived cell cultures isolation was described by Mancini et al, 2011. All cell cultures experiments were approved by the Sant'Andrea Hospital Ethics Committee 2010 (504/10). All samples were obtained upon informed consent of patients.

### Cell proliferation and clonogenic assays

Cell proliferation were quantified using MTT (3-(4,5-dimethylthiazol-2-yl)-2,5-diphenyltetrazolium bromide). Depending on the cell cultures, a variable number of 1000-5000 cells/well were plated in 96 well plates in RPMI 10% FBS with various concentrations of gefitinib. After 96 hrs MTT was added for 4 hrs at 37°C at each well. Formazan crystals formed were dissolved with DMSO. Optical density was measured at 570 and 690 nm with ELISA reader (Thermo Scientific). Experiments were performed in triplicates, the IC50 values were obtained using Kaleidagraph software.

For clonogenic assays single cell suspensions were plated at 2000-5000 cells/well in 24 well/plates, in the presence or absence of various concentration of gefitinib, erlotinib or lapatinib and/or A3 at 25 μg/ml. After 10-15 days colonies were visualized with crystal violet in 20% methanol. Colonies were counted manually, dissolved in methanol and read at the OD595 nm.

### Annexin V binding assay

Annexin V binding assay was performed to quantify apoptosis in cells treated with gefitinib and/or A3. Briefly, 1 × 10^6^ cells were treated for 72 hrs with A3 at 50 μg/ml and gefitnib at variable concentration. After the incubation period, cells were washed in PBS and stained with 5 μl of Annexin V-FITC (Invitrogen) and PI at 5 μg/ml (Invitrogen) for 15 minutes at room temperature in the dark. Apoptosis were determined using MACSquant cytofluorometer. Both early (Annexin V-positive, PI negative) and late (Annexin V-positive, PI positive) apoptotic cells were included in cell death determination.

### Cell cycle Analysis

Cell cycle analysis was performed in cells treated or not with A3 at 50 μg/ml and or gefitinib at 1 μM for using Cell cycle kit from Millipore, according to the manufactures' instructions.

### Immunofluorescence analysis

To evaluate cell proliferation, cells were grown on coverslips and treated with A3 and/or gefitinib and stimulated or not with HRG. After 24 hrs of incubation cells were fixed with 4% paraformaldehyde in PBS followed by treatment with 0.1 M glycine 0.1% Triton X-100 for permeabilization. Incubation with anti-Ki67 antibody and secondary goat anti-rabbit IgG-Texas Red. Nuclei were visualized with DAPI Percentage of Ki67-positive cells was obtained counting for each treatment a total of 500 cells, randomly observed in 10 microscopic fields from three different experiments. Results have been expressed as mean values ± standard errors (SE). p values were calculated using Student's t test and significance level has been defined as p<0.05. Fluorescence was visualized with ApoTome System (Zeiss, Oberkochen, Germany) connected with an Axiovert 200 inverted microscope (Zeiss); image analysis was then performed by the Axiovision software and 3D reconstruction. The mean ± standard error (SE) percent of colocalization was calculated analyzing a minimum of 50 cells for each treatment randomly taken from three independent experiments.

### Western blot analysis

Western was performed using NuPage Bis-Tris electrophoresis system (Invitrogen). The total cellular sample were washed twice in cold PBS and lysed in RIPA buffer (Sigma). The protein concentration was determined using BCA (Pierce). The total cellular protein extracts were separated by SDS-PAGE and transferred to nitrocellulose membrane. Membranes were blocked with 5% milk or 5% BSA in 1X TBS containing 0,05% Tween 20 for 1 hr and incubated with horseradish peroxidase-conjucated antibodies (Sigma Aldrich). Signals were visualized with enhanced chemiluminescent reagent (Amersham).

### DNA extraction and mutational analysis

Genomic DNA from cancer cell lines was isolated using the DNeasy Blood & Tissue kit (Qiagen) and analysed by 0.8% agarose gel electrophoresis to evaluate the DNA quality. The DNA quantity was assessed by using the Nanovue (GE Healthcare) and the purity was evaluated by calculating the 260/280 ratio. Mutations in exon 19, 20 and 21 were assessed by direct sequencing of the PCR product. Methods and primers are available on request.

### Real time RT-PCR Analysis

Total RNA was extracted using TRIzol® reagent (Invitrogen). 200 μg of RNA was treated with DNAse I (Invitrogen) and reverse-transcribed with Superscript™ first strand synthesis system (Invitrogen) according to the manufacturer's instructions. Real-time PCR was carried out with SYBR green (Applied Biosystem). Relative expression levels for p21 were determined by real-time RT-PCR from a standard curve of serial dilutions of cDNA samples and were normalized to 18S expression.

The primers used were:

p21 Forward: 5'-GCAGACCAGCTGACAGATTT
p21 Reverse: 5'-GGATTAGGGCTTCCTCTTGGA
18S Forward: 5'-AACCCGTTGAACCCCATT
18S Reverse: 5'-CCATCCAATCGGTAGTAGCG

### In vivo efficacy studies

All studies have been performed in accordance with “Directive 86/609/EEC on the protection of Animals used for Experimental and other scientific purposes” and made effective in Italy by the Legislative Decree DL 116/92. 6-weeks old NOD/SCID mice (Charles River Laboratories, Inc.) were utilized. After 1 week of acclimatation they were housed five to a plastic cage and fed on basal diet (4RF24, Mucedola S.r.l.) with water *ad libitum*, in an animal facility controlled at a temperature of 23 ± 2°C, 60 ± 5% humidity, and with a 12 h light and dark cycle. All animal protocols used for this study were reviewed and approved by BIOGEM Comitato Etico per la Sperimentazione Animale (CESA) on 26 February 2010. Pe e/10 tumor xenograft have been obtained by serially passaging tumors from previously subcutaneously injected immunodeficient mice. Secondary xenograft have been generated by injecting 1×10^6^ cells/mouse obtained by enzymatic dissociation of previously implanted xenograft. Cell were resuspended in a 50% RGF matrigel (BD Biosciences) solution in PBS and injected in the right flank of the mice in 100 μl volume. Treatment started when tumors reached a 100 mm^3^ volume and mice were allocated five per group. Mice were treated with vehicle (1%Tween80/1%beta-HPCD) or Gefitinib at 100 mg/kg, or A3 in PBS at 20 mg/kg, or combination of the two. Gefitinib was dosed p.o., daily, 5 days/week, while A3 antibody was dosed i.p., once per week and treatment lasted for 4 weeks. At the end of the treatment mice were sacrificed and, after harvesting, tumor weight were determined.

## Supplementary Figures


