# Supplementary material for: Combination therapy with anti-ErbB3 monoclonal antibodies and EGFR TKIs potently inhibits Non-small Cell Lung Cancer
Source: Oncotarget. 2013 Jul 21;4(8):1253–65. doi: 10.18632/oncotarget.1141 (PMC3787155; doi:10.18632/oncotarget.1141)
Supplement: Supplementary file 1 [file oncotarget-04-1253-s001.pdf]

Combination therapy with anti-ErbB3 monoclonal antibodies and EGFR TKIs potently inhibits Non-small Cell Lung Cancer – Noto et al

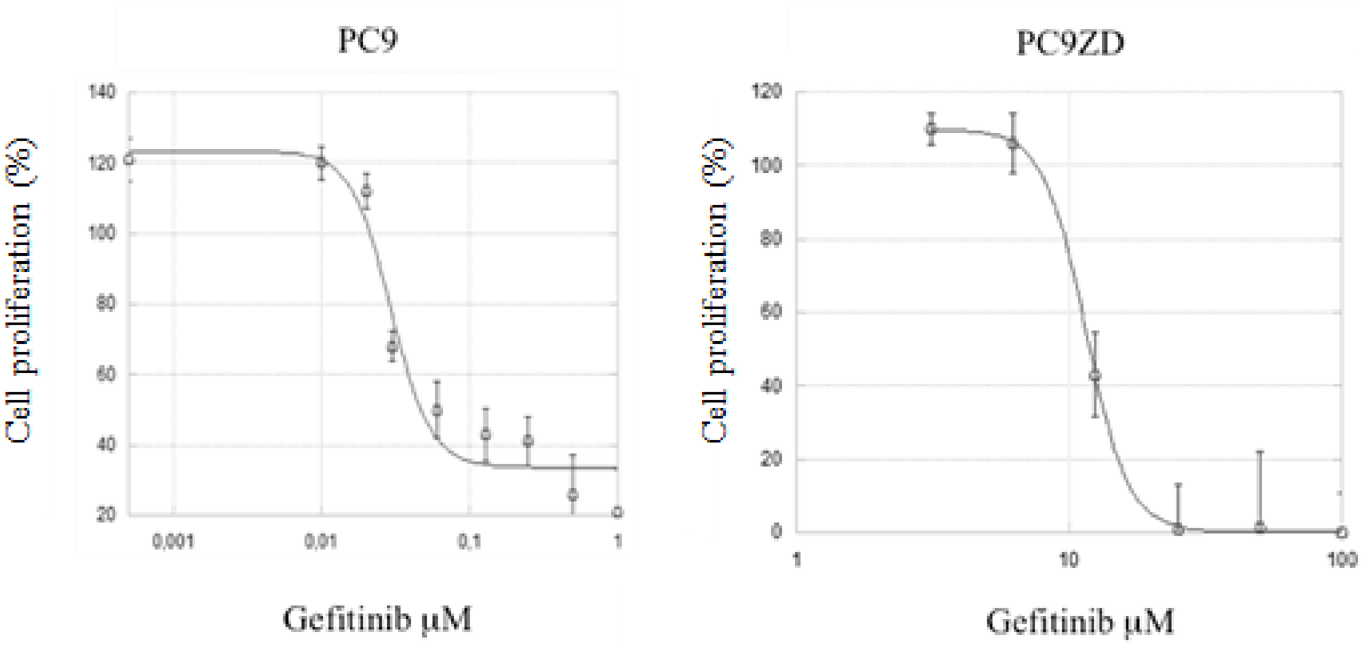

Supplementary Figure 1: Gefitinib sensitivity in PC9 and PC9ZD cell lines. Cells proliferation in PC9 and PC9ZD treated with gefitinib was determined using MTT after 72 hrs of treatment. All bars in the graphs indicate mean values with ±SDs, n=3.

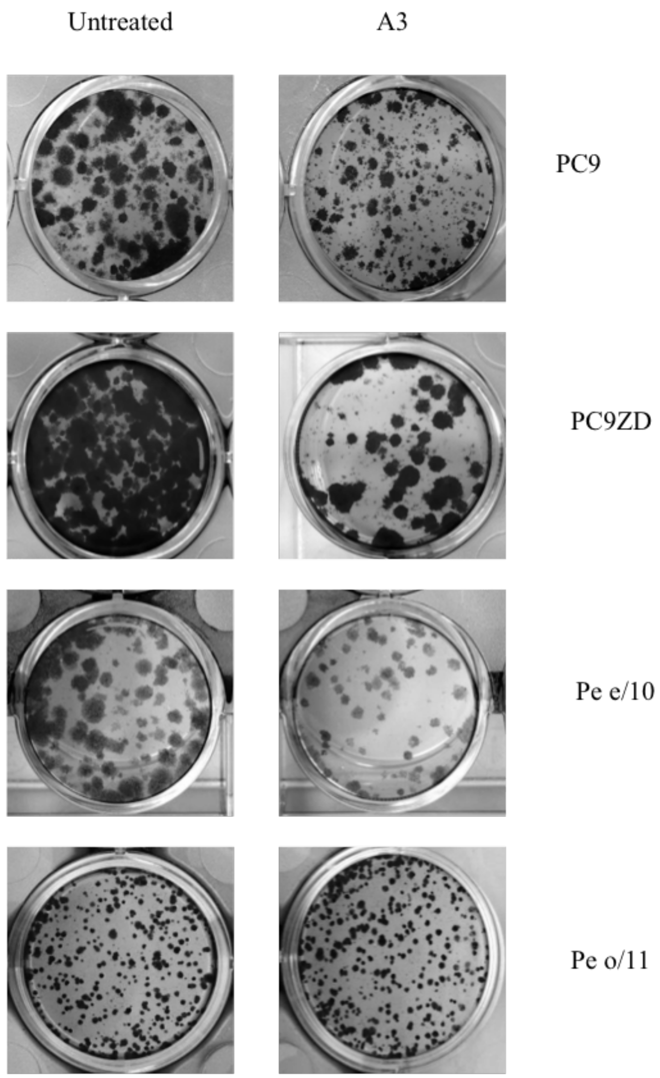

Supplementary Figure 2: Anti-ErbB3 monoclonals reduce the colony forming ability in high ErbB3 lung cancer cell cultures. Representative picture of clonogenic assay performed in PC9, PC9ZD, Pe e/10 and Pe o/11. Cells proliferation was determined after 8-10 days of treatment with 50 µg/ml of A3.

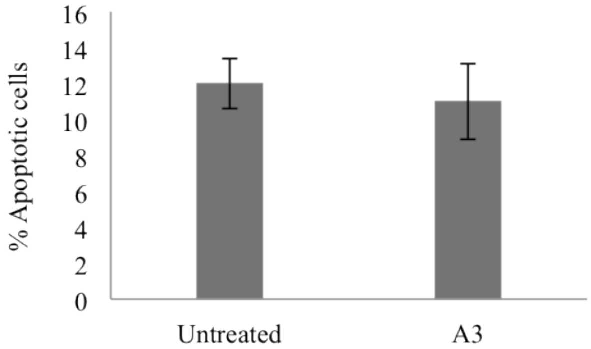

Supplementary Figure 3: Low ErbB3 expressing cell culture does not undergo apoptosis after treatment with A3. Pe o/11 was incubated for 72 hrs with or without the presence of 50 µg/ml of A3. Cells were stained with Annexin V and analyzed for apoptosis induction by FACS analysis. All bars in the graphs indicate mean values with ±SDs of three independent experiments.

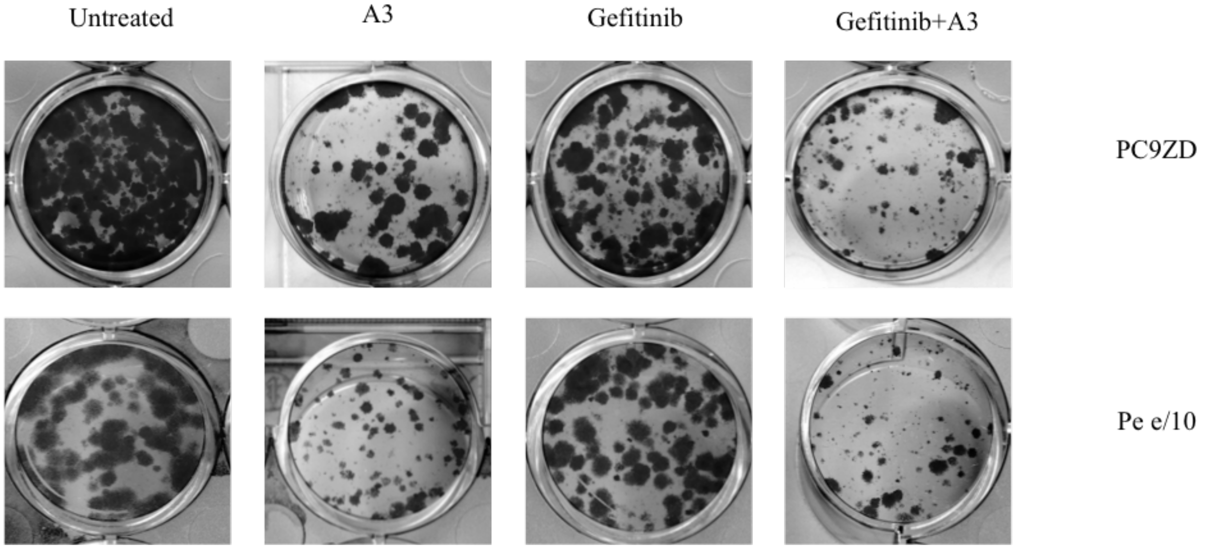

Supplementary Figure 4: Gefitinib sensitivity is increased in A3 treated cells. Picture of a clonogenic assay performed in PC9ZD and Pe e/10 cell cultures treated or not with gefitinib and A3 for 8-10 days, as described in material and methods.

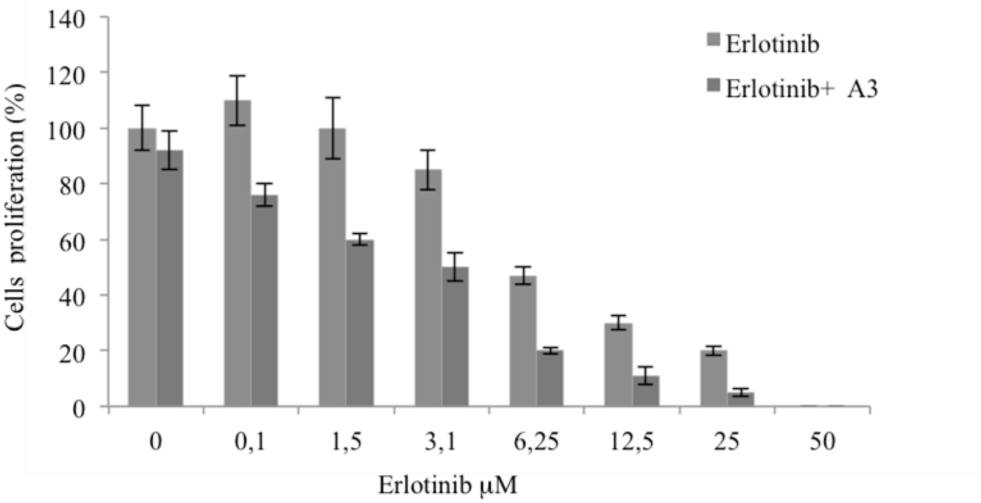

Supplementary Figure 5: Erlotinib sensitivity is increased in PC9ZD incubated with anti-ErbB3 monoclonals. Cells proliferation rate was determined in PC9ZD cell culture treated with the indicated compounds as described previously. Results are the mean ± SDs of three independent experiments.

A

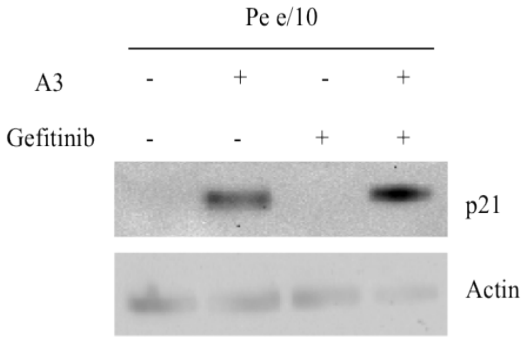

B

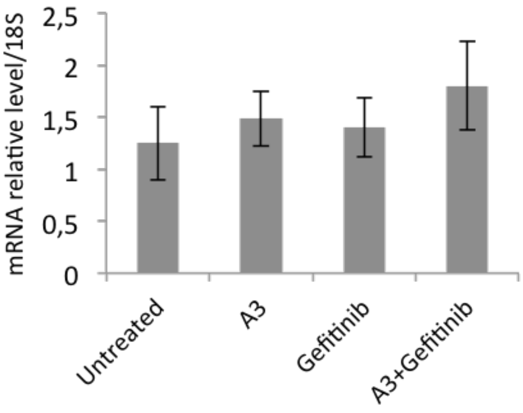

Supplementary Figure 6: A3 induces p21 stabilization of the protein. (a) Western blot analysis of p21 protein level, carried out in Pe e/10 cell culture treated or not with A3 (µg/ml) and or gefitinib (1µM) for 24 hrs. (b) Real time RT-PCR was performed to evaluate the mRNA levels of p21 with respect to housekeeping gene 18S. All bars in the graphs indicate mean values with ±SDs, of three independent experiments.
